# Three new species of the myrmecophilous genus *Doryloxenus* from China (Coleoptera, Staphylinidae, Aleocharinae)

**DOI:** 10.3897/zookeys.456.8584

**Published:** 2014-11-21

**Authors:** Xiao-Bin Song, Li-Zhen Li

**Affiliations:** 1 Department of Biology, College of Life and Environmental Sciences, Shanghai Normal University, 100 Guilin Road, Xuhui District, Shanghai 200234, P. R. China

**Keywords:** Pygostenini, *Doryloxenus*, myrmecophilous, army ant, new species, China

## Abstract

Three new species of the pygostenine genus *Doryloxenus* Wasmann, *viz.*, *Doryloxenus aenictophilus* **sp. n.** (from Zhejiang), *Doryloxenus tangliangi* **sp. n.** (from Zhejiang), and *Doryloxenus songzhigaoi* **sp. n.** (from Yunnan), are described, illustrated and distinguished from the Asian congeners. An identification key to the Chinese species is given.

## Introduction

The myrmecophilous genus *Doryloxenus* Wasmann currently contains 36 species worldwide (Jacobson and Kistner 1975; Jacobson 1980; Naomi 1996; Pace 1998; Kistner et al. 2003; Assing 2009), among which three are known from China: *Doryloxenus hongkongensis* Pace (Hongkong), *Doryloxenus rougemonti* Pace (Hongkong), and *Doryloxenus yunnanus* Assing (Yunnan). Members of *Doryloxenus* are commonly found in association with the army ant genus *Dorylus*, but symbiotic hosts of all Chinese *Doryloxenus* remain unknown.

In 2013, the senior author and his colleagues surveyed the myrmecophilous and termitophilous staphylinidae at Longwangshan Natural Reserve, Zhejiang (Fig. 4A) and Zizhi Village, Yunnan (Fig. 4C), and collected a series of aleocharine beetles from the colonies of army ant *Aenictus* sp. and *Dorylus orientalis*. A closer examination of this material revealed three new species of the genus *Doryloxenus*, which are described herein.

## Material and methods

Holotypes and most of the paratypes are deposited in the Insect Collection of the Shanghai Normal University, Shanghai, China (SNUC), and some of paratypes are deposited in the Kyushu University Museum, Fukuoka, Japan (KUM).

Specimens were killed with ethyl acetate and preserved in 75% ethanol before dissection; photos of habitus were taken by a Canon EOS 7D with an MP-E 65mm macro photo lens; photos of characteristic pattern were taken by a Canon G9 Camera mounted on an Olympus CX31 microscope.

The following abbreviations are applied in the text: BL – body length, from the anterior margin of the head to the posterior margin of the abdominal tergite VIII; FBL – forebody length, from the clypeal anterior margin to the posterior margin of elytra; HL – head length, from the clypeal anterior margin to the occipital constriction; PL – length of the pronotum along the midline; HW – width of the head across the eyes; PW – maximum width of the pronotum.

## Taxonomy

### Doryloxenus

Taxon classificationAnimaliaColeopteraStaphylinidae

Wasmann

Doryloxenus Wasmann, 1898: 101 (original description, type species: *Doryloxenus cornutus* Wasmann, 1898); Jacobson and Kistner 1975: 299 (key, diagnosis).

#### Remarks.

The genus is most similar to *Odontoxenus* Kistner in general appearance. It can be easily separated from *Odontoxenus* by the eyes having no part of their surface on the anterior margin of the head, the quadrate mesocoxal cavity, and the shorter mesosternum (Jacobson and Kistner 1975). *Doryloxenus* is also similar to *Pygoplanus* Kistner by the limuloid shape, but can be distinguished from it by the different shapes of the mandibles and labrum, the maxillary palpus distinctly longer than the setulate galea (Kistner et al. 2003).

### Doryloxenus aenictophilus sp. n.

Taxon classificationAnimaliaColeopteraStaphylinidae

http://zoobank.org/0499D286-4426-4DF2-A379-02BC548E288F

Fig. 1

#### Type material.

**Holotype: China:** ♂, labeled ‘CHINA: Zhejiang Province, Huzhou City, Anji County (安吉县), Longwangshan (龙王山), alt. 1330m, 30°24'15.53"N, 119°26'36.81"E, 14-V-2013, X.-B. Song leg., from a colony of *Aenictus* sp. / HOLOTYPE [red], *Doryloxenus aenictophilus* sp. n., Song & Li det. 2014, SNUC’. **Paratype: China:** 1♂, 1♀, 8 sex?, same data as holotype, bearing the following label: ‘PARATYPE [yellow], *Doryloxenus aenictophilus* sp. n., Song & Li det. 2014’. (SNUC, KUM).

#### Comparative notes.

*Doryloxenus aenictophilus* is most similar to *Doryloxenus tangliangi* described below by the forebody sparsely covered with yellow setae and the macrochaetotaxy of tergites II–V: 6, 4, 4, 4, 4. It differs from *Doryloxenus tangliangi* by the smaller eyes, the shorter elytra and the reduced hind wings. The new species is also similar to the unique blind and wingless species *Doryloxenus coecus* Kistner by the light color and the short elytra, but can be easily distinguished from it by the presence of small eyes, the different macrochaetotaxy of abdominal tergites II–VIII.

#### Description.

**Figure 1.**
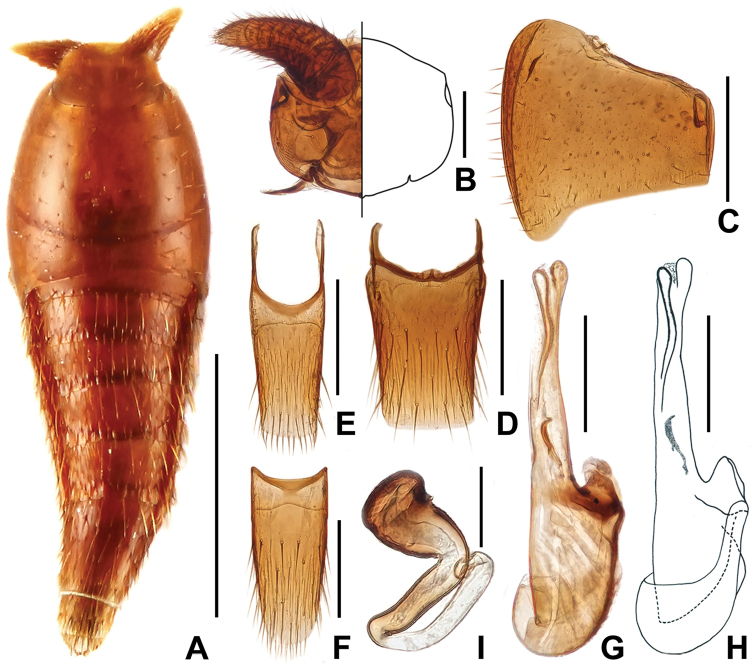
*Doryloxenus aenictophilus* sp. n. **A** habitus **B** head **C** Elytron **D** tergite VII **E** tergite VIII **F** sternite VIII **G** median lobe of aedeagus, in lateral view **H** ditto **I** spermatheca. Scales (mm): **A** = 0.5; **B, G, H** = 0.1; **C, F** = 0.2; **I** = 0.05.

Body (Fig. 1A) smooth, glossy. Coloration: Light reddish-brown overall.

Head shaped as in Fig. 1B, sparsely covered with yellow setae; eyes small. Pronotum (Fig. 1A) wider than long, about 1.44 times as wide as long; disc sparsely covered with yellow setae. Elytra (Fig. 1A, C) short, wider than long, about 3.72 times as wide as long; disc sparsely covered with yellow setae, with a row of setae on lateral margins. Hind wings reduced. Abdomen wedge-shaped; posterior margins of tergite II–VI with a row of very long yellowish setae; abdominal tergite VII (Fig. 1D) truncate at apex, with 2 pairs of macrochaetae at the anterior 1/3; tergite VIII (Fig. 1E) slightly truncate at apex, with 1 pair of lateral macrochaetae; sternite VIII shaped as in Fig. 1F. Macrochaetotaxy of abdominal tergites II–VIII: 6, 4, 4, 4, 4, 4, 2.

Male. Median lobe of aedeagus shaped as in Fig. 1G–H.

Female. Spermatheca with apical part strongly swollen, shaped as in Fig. 1I.

#### Measurements.

BL: 1.48–1.61; FBL: 0.64–0.68; PL: 0.33–0.35; PW: 0.48–0.51; PW/PL: 1.42–1.45; HW/PW: 0.51–0.56.

#### Distribution.

East China: Zhejiang.

#### Symbiotic host.

*Aenictis* sp. (Fig. 5A–B). According to the key provided by Jaitrong and Yamane (2011), the host ant should belong to the *Aenictis ceylonicus* group. This is the first record of a *Doryloxenus* associated with *Aenictus* ant together with the next new species.

#### Biological notes.

Eleven *Doryloxenus aenictophilus* were sifted together with a large series of *Doryloxenus tangliangi* from the colony of *Aenictus* sp. nesting under a rock. One individual was observed riding on the head of a worker ant.

#### Etymology.

The specific name is a combination of ‘*Aenictus*’, generic name of the ant host, and the Greek stem ‘*philos*’, meaning ‘to be fond of’.

### Doryloxenus tangliangi sp. n.

Taxon classificationAnimaliaColeopteraStaphylinidae

http://zoobank.org/BBD8E915-9ED8-4361-B3E1-0F500BB2BAD2

Fig. 2

#### Type material.

**Holotype: China:** ♂, labeled ‘CHINA: Zhejiang Province, Huzhou City, Anji County (安吉县), Longwangshan (龙王山), alt. 1330m, 30°24'15.53"N, 119°26'36.81"E, 14-V-2013, X.-B. Song leg., from a colony of *Aenictus* sp. / HOLOTYPE [red], *Doryloxenus tangliangi* sp. n., Song & Li det. 2014, SNUC’. **Paratype: China:** 5♂, 3♀, 102sex?, same data as holotype, bearing the following label: ‘PARATYPE [yellow], *Doryloxenus tangliangi* sp. n., Song & Li det. 2014’. (SNUC, KUM).

#### Comparative notes.

*Doryloxenus tangliangi* is most similar to *Doryloxenus aenictophilus* described above by the forebody sparsely covered with yellow setae and the macrochaetotaxy of tergites II–V: 6, 4, 4, 4, 4. It differs from *Doryloxenus aenictophilus* by the larger eyes, the relatively long elytra, as well as the different shapes of the aedeagus and spermatheca.

#### Description.

**Figure 2.**
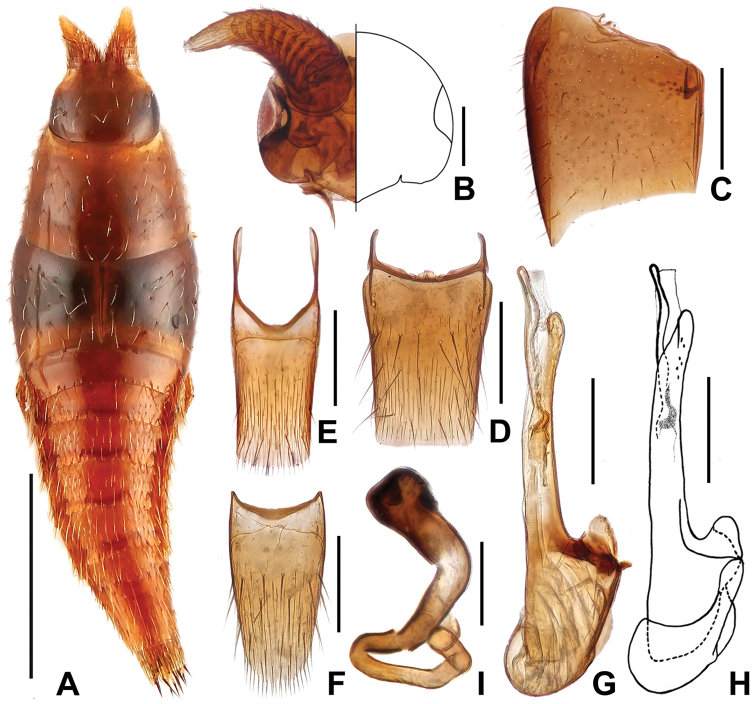
*Doryloxenus tangliangi* sp. n. **A** habitus **B** head **C** Elytron **D** tergite VII **E** tergite VIII **F** sternite VIII **G** median lobe of aedeagus, in lateral view **H** ditto **I** spermatheca. Scales (mm): **A** = 0.5; **B, G, H** = 0.1; **C, F** = 0.2; **I** = 0.05.

Body (Fig. 2A) smooth, glossy. Coloration: Light reddish-yellow overall.

Head shaped as in Fig. 2B, sparsely covered with long yellowish setae; eyes large. Pronotum (Fig. 2A) wider than long, about 1.44 times as wide as long; disc sparsely covered with long yellowish setae. Elytra (Fig. 2A, C) about 2.73 times as wide as long; disc sparsely covered with long yellowish setae, with a row of setae on lateral margins. Abdomen wedge-shaped; posterior margins of tergite II–VI with a row of very long yellowish setae; abdominal tergite VII (Fig. 2D) truncate at apex, with 2 pairs of macrochaetae at the anterior 1/3 and 3 pairs near apex; tergite VIII (Fig. 2E) slightly truncate at apex, with 1 pair of lateral macrochaetae and 2 pairs near apex; sternite VIII shaped as in Fig. 2F. Macrochaetotaxy of abdominal tergites II–VIII: 6, 4, 4, 4, 4, 10, 6.

Male. Median lobe of aedeagus shaped as in Fig. 2G–H.

Female. Spermatheca with apical part strongly swollen, shaped as in Fig. 2I.

#### Measurements.

BL: 1.70–1.93; FBL: 0.77–0.86; PL: 0.34–0.37; PW: 0.51–0.52; PW/PL: 1.38–1.53; HW/PW: 0.63–0.67.

#### Distribution.

East China: Zhejiang.

#### Symbiotic host.

*Aenictus* sp. (Fig. 5A–B).

#### Biological notes.

Most of the specimens were sifted from a colony of *Aenictus* ant, at least four individuals were observed riding on the abdomen of worker ants (Fig. 4B).

#### Etymology.

Dedicated to Dr. Liang Tang, who found the colony of the host ants.

### Doryloxenus songzhigaoi sp. n.

Taxon classificationAnimaliaColeopteraStaphylinidae

http://zoobank.org/CFB4FD49-5CA3-4DAD-97F5-BB77C22FA480

Fig. 3

#### Type material.

**Holotype: China:** ♂, labeled ‘CHINA: Yunnan, Tengchong City, Mingguang Town (明光乡), Zizhi Vill. (自治村), Donghe (东河), alt. 2400m, 25°42'57"N, 98°35'42"E, 30-IV-2013, X.-B. Song leg., from a colony of *Dorylus orientalis* / HOLOTYPE [red], *Doryloxenus songzhigaoi* sp. n., Song & Li det. 2014, SNUC’. **Paratype: China:** 3♂, same data as holotype, bearing the following label: ‘PARATYPE [yellow], *Doryloxenus songzhigaoi* sp. n., Song & Li det. 2014’. (SNUC).

#### Comparative notes.

*Doryloxenus songzhigaoi* is most similar to *Doryloxenus nepalensis* Naomi in general appearance, but can be easily separated from it by the different macrochaetotaxy of abdominal tergites II–VIII. The new species can be distinguished from the other congener known from the Gaoligong Shan, *Doryloxenus yunnanus*, by the slender tergite VIII, rounded apex of sternite VIII, and different shape of the aedeagal median lobe.

#### Description.

**Figure 3.**
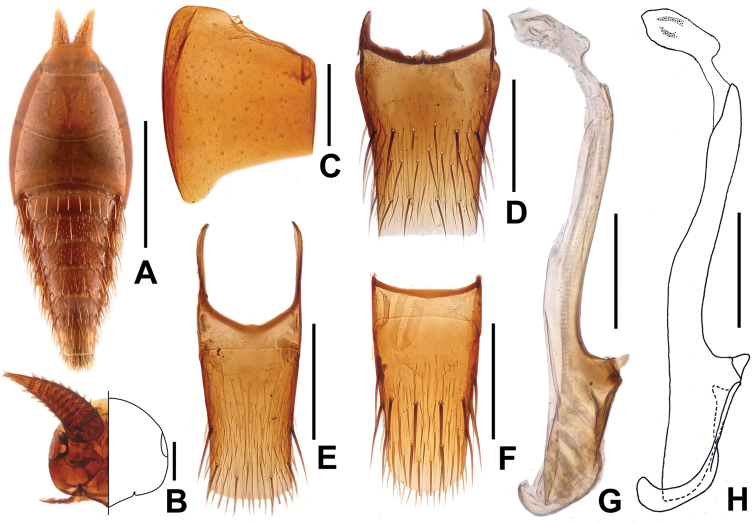
*Doryloxenus songzhigaoi* sp. n. **A** habitus **B** head **C** Elytron **D** tergite VII **E** tergite VIII **F** sternite VIII **G** median lobe of aedeagus, in lateral view **H** ditto. Scales (mm): **A** = 0.5; **B, G, H** = 0.1; **C, F** = 0.2.

**Figure 4.**
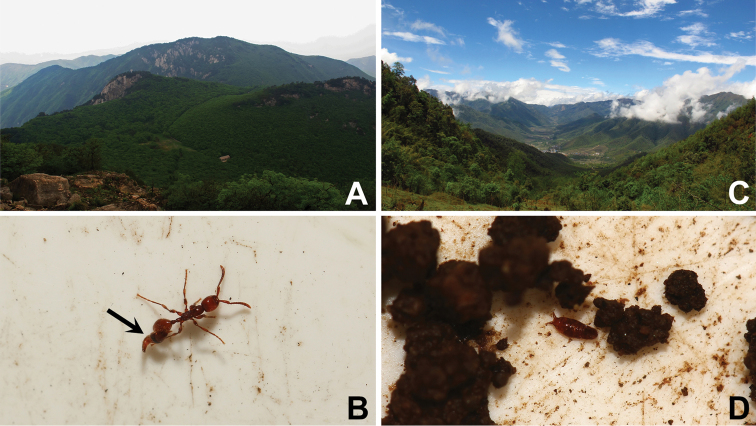
**A** Longwangshan Natural Reserve **B** *Doryloxenus tangliangi* riding on the abdomen of an *Aenictus* ant **C** Type locality of *Doryloxenus songzhigaoi* (Donghe, Zizhi Village) **D** *Doryloxenus songzhigaoi*, habitus.

Body (Figs 3A, 4D) smooth, glabrous. Coloration: Light reddish-yellow overall.

Forebody shaped as in Fig. 3A, with sparse punctation. Head shaped as in Fig. 3B. Pronotum (Fig. 3A) about 1.71 times as wide as long. Elytra (Fig. 3A, C) about 2.55 times as wide as long. Abdomen wedge-shaped; posterior margins of tergite II–VI with a row of very long yellowish setae; abdominal tergite VII (Fig. 3D) truncate at apex, with 2 pairs macrochaetae at middle and 3 pairs near apex; tergite VIII (Fig. 3E) slightly truncate at apex, with 1 pair of lateral macrochaetae and 2 pairs near apex; sternite VIII shaped as in Fig. 3F. Macrochaetotaxy of abdominal tergites II–VIII: 4, 2, 4, 4, 4, 10, 6.

Male. Median lobe of aedeagus shaped as in Fig. 3G–H.

Female. Unknown.

#### Measurements.

BL: 1.51–1.61; FBL: 0.69–0.72; PL: 0.31–0.33; PW: 0.53–0.56; PW/PL: 1.66–1.74; HW/PW: 0.56–0.64.

#### Distribution.

Southwest China: Yunnan.

#### Symbiotic host.

*Dorylus orientalis* Westwood, 1835.

#### Etymology.

Named after Mr. Zhi-Gao Song, the senior author’s father.

### Key to the species of *Doryloxenus* from China

**Table:** 

1	Forebody glabrous, with sparse and fine punctation	**2**
–	Forebody sparsely covered with yellow setae	**5**
2	Body broad; abdominal tergites and paratergites sparsely covered with setae	**3**
–	Body slender; abdominal paratergites without setae. (Hongkong)	***Doryloxenus hongkongensis* Pace**
3	Posterior margin of elytra slightly concave	**4**
–	Posterior margin of elytra truncate. (Hongkong)	***Doryloxenus rougemonti* Pace**
4	Abdominal sternite VIII rounded at apex; aedeagus shaped as in Fig. 3G–H. (Yunnan)	***Doryloxenus songzhigaoi* sp. n.**
–	Abdominal sternite VIII slightly truncate at apex; aedeagal distal crest well developed, apical lobe curved ventrad at the middle. (Yunnan)	***Doryloxenus yunnanus* Assing**
5	Eyes generalized in size; elytra relatively long; hind wings. (Zhejiang)	***Doryloxenus tangliangi* sp. n.**
–	Eyes small; elytra short; hind wings reduced. (Zhejiang)	***Doryloxenus aenictophilus* sp. n.**

**Figure 5.**
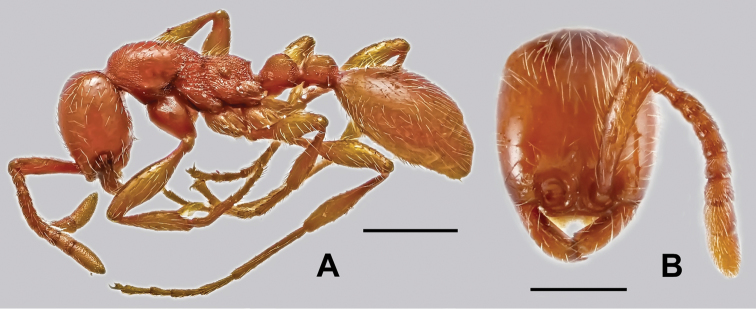
Host ant of *Doryloxenus aenictophilus* and *Doryloxenus tangliangi* **A** *Aenictus* sp., in lateral view **B** ditto, head in full face view. Scales (mm): **A** = 0.5; **B** = 0.3.

## Supplementary Material

XML Treatment for Doryloxenus

XML Treatment for Doryloxenus aenictophilus

XML Treatment for Doryloxenus tangliangi

XML Treatment for Doryloxenus songzhigaoi
